# Nanoscale Imaging of Palladium-Enhanced Photocatalytic Reduction of 4-Nitrothiophenol on Tungsten Disulfide Nanoplates

**DOI:** 10.1021/acs.nanolett.4c03702

**Published:** 2024-10-07

**Authors:** Swati J. Patil, Dmitry Kurouski

**Affiliations:** † Department of Biochemistry and Biophysics, Texas A&M University, College Station, Texas 77843, United States; ‡ Department of Biomedical Engineering, Texas A&M University, College Station, Texas 77843, United States

**Keywords:** Tungsten disulfide, palladium, photocatalysis, TERS imaging

## Abstract

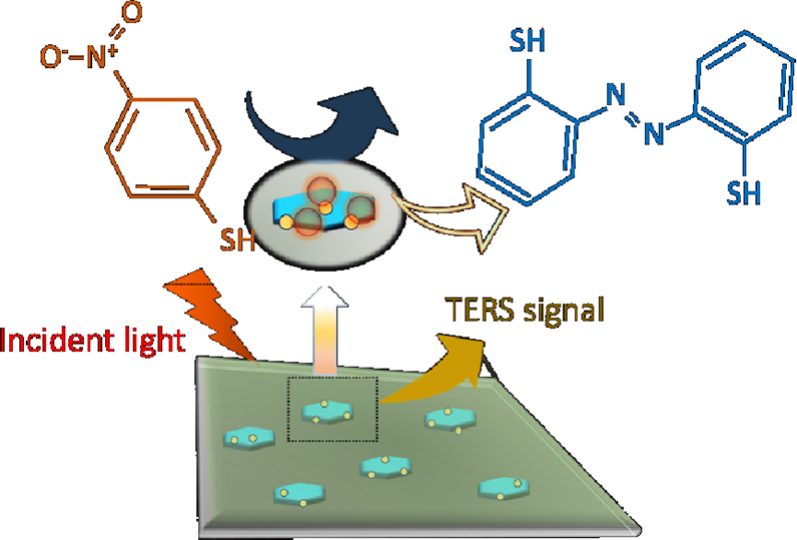

Two-dimensional (2D) dichalcogenides are modern nanomaterials with unique physical and chemical properties. These materials possess band gaps in the infrared and visible regions of the electromagnetic spectrum that can be tuned by their molecular composition. Excitons generated as a result of such light-matter interactions are capable of catalyzing chemical reactions in molecular analytes present on the dichalcogenide surfaces. However, the photocatalytic properties of such nanomaterials remain poorly understood. In the current study, we utilize tip-enhanced Raman spectroscopy (TERS) to examine photocatalytic reduction of 4-nitrothiophenol (4-NTP) to *p,p*′-dimercaptoazobisbenzene (DMAB) on tungsten disulfide (WS_2_) nanoplates and WS_2_ coupled with palladium nanoparticles (WS_2_@PdNPs). Our results indicate that although both WS_2_ and WS_2_@Pd were capable of reducing 4-NTP into DMAB, the metallic hybrid demonstrated much greater yield and rates of DMAB formation compared to WS_2_ nanoplate. These results indicate that coupling of catalytic metals to dichalcogenides could be used to enhance their catalytic properties.

Metallic nanostructures formed by plasmonic and nonplasmonic metals demonstrate strong absorption properties in the visible and infrared parts of electromagnetic spectrum.^1−3^ Light absorption generates coherent oscillations of conductive electrons on their surfaces, also known as localized surface plasmon resonances (LSPRs).^4,5^ LSPRs determine 10^6^–10^8^ enhancement of Raman scattering from molecules present in the close proximity to such nanostructure, a phenomenon known as surface-enhanced Raman scattering.^6,7^ LSPRs can decay producing heat or decay via nonradiative pathways producing hot carriers.^8,9^ These highly energetic short-living species can be directly or indirectly injected into molecular orbitals of molecules present on the metal surfaces and, consequently, trigger chemical transformations.^10,11^ During the past decade, a substantial amount of evidence was accumulated about such plasmon-driven transformations.^12,13^ Furthermore, it was found that coupling of plasmonic nanostructures with catalytic metals, such as platinum (Pt) or palladium (Pd), allows for the substantial expansion of the chemical reactions that could be catalyzed by such bimetallic nanoreactors.^14−18^

During the past decade, substantial advances have been made in the fabrication of two-dimensional (2D) dichalcogenides.^35^ These modern nanomaterials possess unique physical and chemical properties due to simplicity of their fabrication, unique light-metal coupling and tunable band gaps in visible and infrared parts of electromagnetic spectrum.^12,19^ 2D dichalcogenides became broadly used in optoelectronics applications^26^ such as light sources,^27^ optical modulators,^28^ photodetectors,^29^ field-effect transistors,^30^ logic circuits,^31^ sensors^32−34^ etc.^20−22^ Furthermore, 2D dichalcogenides exhibit unique catalysts and optoelectronics properties. For instance, An et al.^25^ found that a MXene-based Au@Ag@Pd/Ti_3_C_2_ photocatalyst could be used for light-driven hydrogenation of nitroaromatics. Lambin and co-workers found that molybdenum disulfide (MoS_2_) flakes could catalyze photocatalytic reduction of 4-nitrobenzenethiol (4-NBT) to *p,p*′-dimercaptoazobisbenzene (DMAB),^19^ a chemical reaction that was previously evident only for plasmonic metals.^20^ Expanding upon this, we utilized tip-enhanced Raman spectroscopy (TERS) to examine catalytic properties of tungsten disulfide (WS_2_) nanoplates, as well as WS_2_ coupled with palladium nanoparticles (WS_2_@Pd). In TERS, scanning probes could be brought in the close proximity to the surface of 2D materials.^1−4^ Next, the apex of the scanning probe is illuminated by a laser light. This generates LSPRs at the metal surface.^5−12^ LSPRs enhance Raman scattering from the molecules located directly under the tip. This provides TERS single-molecule sensitivity.^13,14^ Furthermore, the electric field is confined under the tip down to a pico-volume enabling subnanometer spatial resolution in TERS imaging.^13,21−23^ t

A chemical approach was used for the WS_2_@NPs synthesis. The commercially available stacked tungsten disulfide (WS_2_) nanoplatelets (Sigma-Aldrich) were further modified in ethanolic solution to separate the nanoplates, Figure 1, A. The WS_2_ stacked layers were added to the ethanolic solution and subjected to sonication for 4 h so that 2D WS_2_ suspensions formed. Further, it was centrifuged at 10000 rpm for 15 min in ethanol. The formed 2D dichalcogenides are typically seen as an interlayer of S and W atoms stabilized by van der Waals forces. Next, WS_2_ nanoplates were mixed with PdNPs to synthesize WS_2_@Pd hybrids, Figure 1, A. PdNPs were prepared with ascorbic acid (10 mM) and subsequentially added H_2_PdCl_4_ (5 mM), followed by stirring for 30 s. Further, WS_2_ nanoplates (250 μL) were mixed with the above solution by stirring for 10 s, and the above mixture was kept at room temperature for 30 min until the Pd layer formed on the top of the WS_2_ nanoplates. In the final step, the solution was centrifuged under water and ethanol for 5 min at 5000 rpm, redispersed in ethanol, and sonicated for over 20 s to form the final WS_2_@Pd hybrids. UV–vis spectroscopy revealed that WS_2_ had an absorption band at ∼634 nm, Figure 1, B, whereas WS_2_@Pd exhibited the absorption ∼474 nm, originating from the absorbance of PdNPs evident at 440 nm. The 34 nm red shift points to the coupling between WS_2_ and PdNPs.

**Figure 1:**
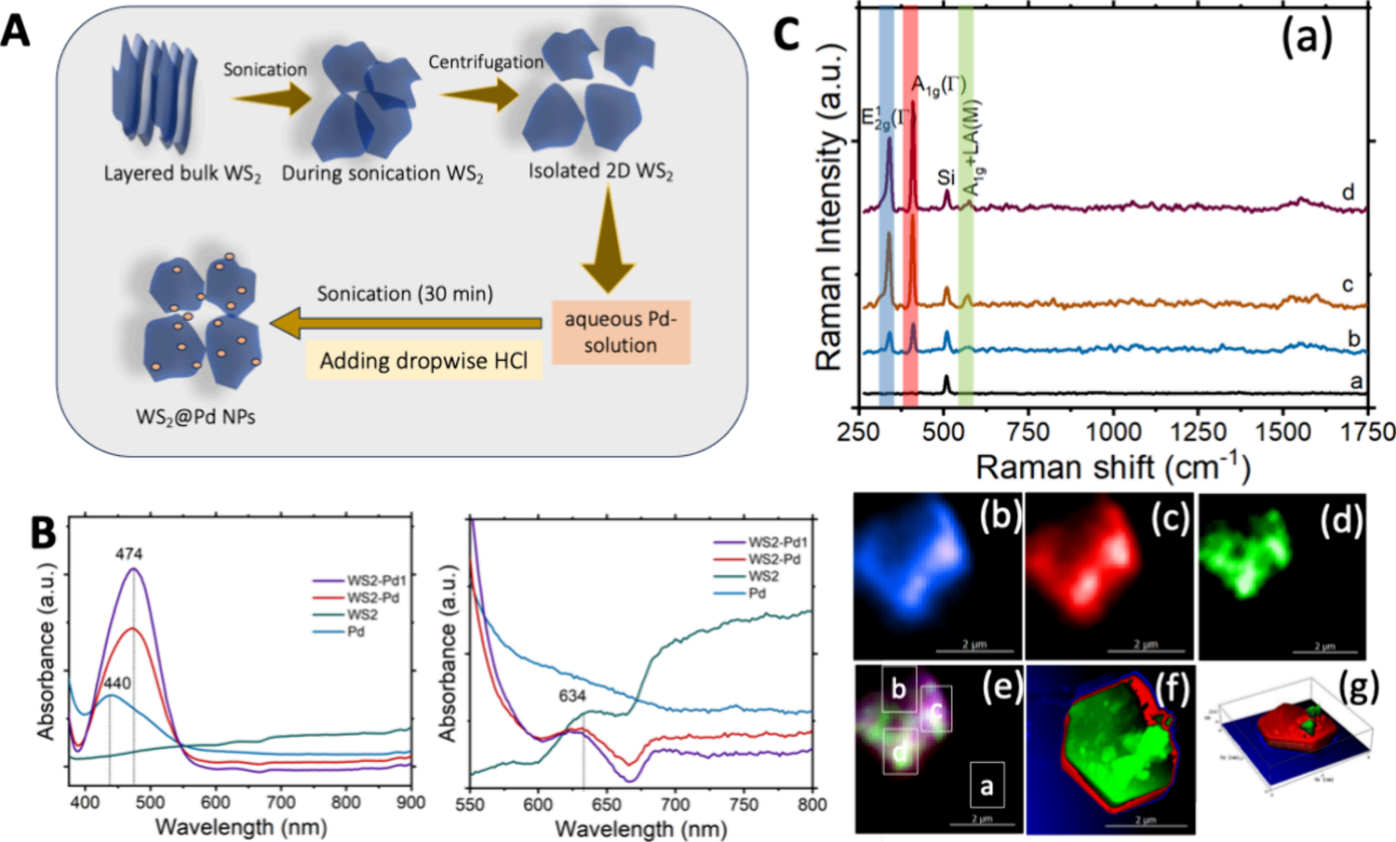
Synthesis and physical properties of WS_2_. A. The scheme of synthetic approaches used for the fabrication of WS_2_@PdNPs; B. UV–visible spectra of WS_2_ and WS_2_@PdNPs; C. (a) TER spectra of the WS_2_ nanoplate. (b-e) TERS image of WS_2_ nanoplate with E^1^_2g_ (blue), A_1g_ (red) and A_1g_+LA(M) (green) bands. (f) TERS image of WS_2_ from overlapping E_2g_, A_1g_ and A_1g_+LA(M) vibrations. (g) Corresponding AFM image of WS_2_ nanoplate.

We also used TERS to examine the crystalline properties of WS_2_. We observed three vibrational bands in the acquired TERS spectra that corresponded to E^1^_2g_ (M) mode at 355 cm^–1^; A_1g_ (τ) mode at 417 cm^–1^ and A_1g_ × LA(M) at 582 cm^–1^, Figure 1, C. The strong in-plane E^1^_2g_ mode originated from the out-of-plane vibrations of S atoms relative to the W atoms. A_1g_ (τ) mode could be assigned to the out-of-plane vibration of S atoms, Figure 1, C, panel (a). The vibrational mode ∼417 cm^–1^ originated from the second-order process that involved the longitudinal acoustic phonons at the M point (LA(M)). Strong intensity of the LA(M) vibration in the acquired TER spectra indicated the multilayered nature of the synthesized WS_2_ nanosheets. TER maps of E^1^_2g_ (blue-pixel), A_1g_ (τ) (green-pixel), and 2 × LA(M) (red-pixel) was displayed in Figure 1, C, panels (b), (c), and (d), respectively and corresponding atomic force microscopy (AFM) image as shown in Figure 1, C, panel (f). We also found that WS_2_@PdNPs exhibited crystalline properties similar to those of WS_2_, Figure S1.

Next, we exposed WS_2_ nanoplates adsorbed onto silicon (Si) wafer to an ethanolic solution of 4-NTP. After several seconds of exposure, the excess of 4-NTP was removed by extensive rinsing of WS_2_ nanoplates with pure ethanol. Next, WS_2_ nanoplates were dried under a nitrogen flow and analyzed using TERS. 4-NTP has a distinct vibrational band at 1069, 1098, 1330, and 1563 cm^–1^ that were observed in TERS spectra collected at different sites of WS_2_ nanoplates, Figure 2. We also found that TERS spectra acquired in the central part of the WS_2_ nanoplates exhibited a doublet at 1437 and 1468 cm^–1^ that could be assigned to DMAB.^24,25^ These results indicate that WS_2_ nanoplates were able to reduce 4-NTP into DMAB. Although the exact mechanism of DMAB formation on WS_2_ is unclear, our previous study suggests that high reactivity of NO_2_ group was determined by its low photopotential.^26^ Density functional theory calculations also showed that 4-NTP would uptake two electrons and two protons from the monolayer of water present on the surface of such nanostructures at ambient conditions.^26^ As a result, the NO_2_ group would be reduced to NO and then to NHOH. However, the following reduction of NHOH to NH_2_ is thermodynamically unfavorable compared to dimerization of two molecules that possess NHOH groups into the molecule with one azo bond (DMAB).

**Figure 2:**
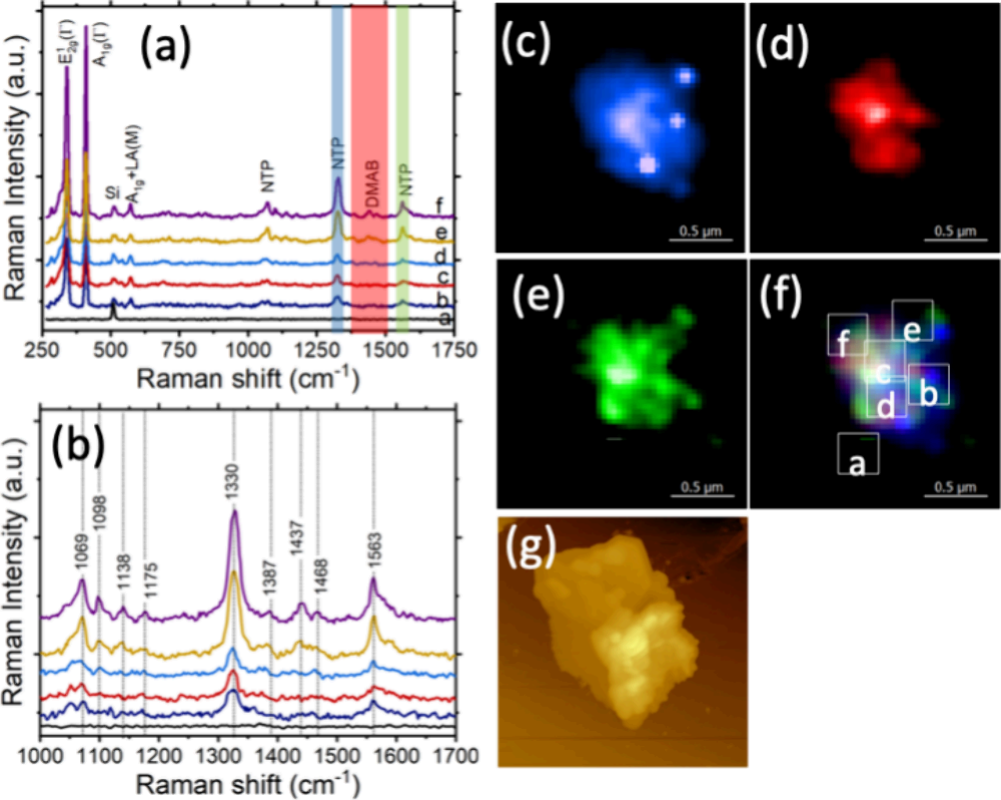
Catalytic reactivity of WS_2_ nanoplates. (a) TER spectra extracted from chemical maps on 4-NTP decorated WS_2_ nanoplate: (b) showing presence of 4-NTP and DMAB. (c–f) TERS image of WS_2_ with (c) and (e) 4-NTP and (d) DMAB. (f) TERS image of WS_2_ from overlapping 4-NTP and DMAB. Intensity of 1330 and 1563 cm^–1^ band of 4-NTP is shown in blue and green; and intensity of 1387, 1437, and 1468 cm^–1^ band (azo vibration) of DMAB is shown in red. (g) Corresponding AFM image of WS_2_. The scanning step size was 10 nm per pixel, spectral acquisition time was 0.5 s.

Our results also indicate that reactivity of the central parts of WS_2_ nanoplates was greater than the reactivity at the edges of these nanomaterials. One can expect that a scanning probe exposed to the nanostructure surfaces could be responsible for DMAB formation. To verify this hypothesis, we performed nano-infrared analysis of WS_2_ nanoplates with 4-NTP on their surface. Nano-IR imaging confirmed the presence of DMAB (1435 and 1465 cm^–1^) on the surface of WS_2_ nanoplates that were never explored by TERS, as shown in Figure S2.

TERS imaging of WS_2_@PdNPs exposed to 4-NTP revealed a much greater yield of DMAB on their surface compared to WS_2_, Figure 3. This conclusion could be made by very strong intensities of 1431 and 1466 cm^–1^ that could be assigned to DMAB in TERS spectra acquired from various parts of WS_2_@PdNPs, Figure 3. Similar to WS_2_, we found that central parts of WS_2_@Pd nanoplates exhibited greater intensity of DMAB vibrational bands compared to the edges of WS_2_@Pd hybrids. These results show that central parts of WS_2_@Pd have greater photocatalytic reactivity compared to edges of WS_2_@PdNPs. This observation could be explained by the higher density of PdNPs in the central parts of these nanostructures. Although PdNPs alone could not catalyze the dimerization of 4-NTP into DMAB, the coupling of PdNPs with WS_2_ yielded a highly efficient catalyst in which PdNPs played a critically important role.

**Figure 3:**
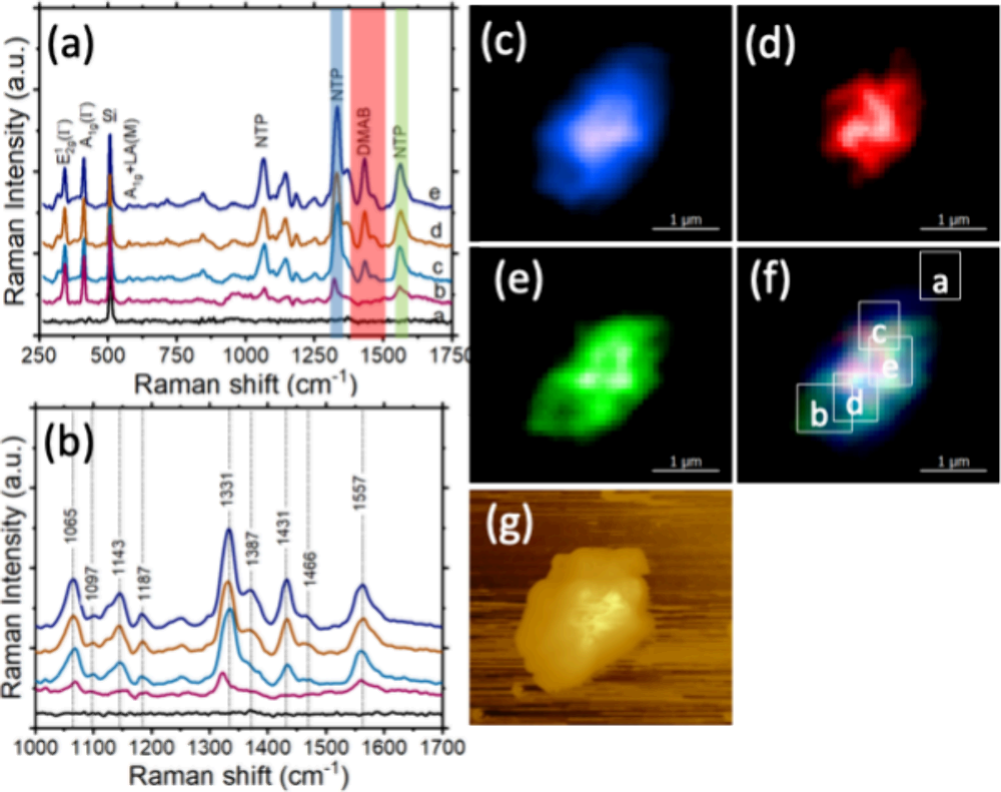
(a) Catalytic reactivity of WS_2_@PdNPs. TER spectra extracted from chemical maps on 4-NTP decorated WS_2_@PdNPs: (b) showing the presence of 4-NTP and DMAB. (c–f) TERS image of WS_2_ with (c) and (e) 4-NTP and (d) DMAB. (f) TERS image of WS_2_ from overlapping 4-NTP and DMAB. Intensity of 1331 and 1557 cm^–1^ band of 4-NTP is shown in blue and green; and intensity of 1387, 1431, and 1466 cm^–1^ band (azo vibration) of DMAB is shown in red. (g) Corresponding AFM image of WS_2_@PdNPs. The scanning step size was 10 nm per pixel, spectral acquisition time was 0.5 s.

It should be noted that vibrational signatures of DMAB were also evident in the nano-IR spectra acquired from the surface of WS_2_@PdNPs exposed to 4-NTP, as shown in Figure 4. These results indicate that WS_2_@PdNPs rather than the metallic scanning probe used in TERS were capable of photo reducing 4-NTP into DMAB. Similar to TERS, nano-IR revealed a much stronger intensity of 1448 cm^–1^ in the nano-IR spectra acquired from WS_2_@PdNPs to WS_2_. These results indicate that WS_2_@PdNPs enabled much greater photoconversion of 4-NTP into DMAB.

**Figure 4:**
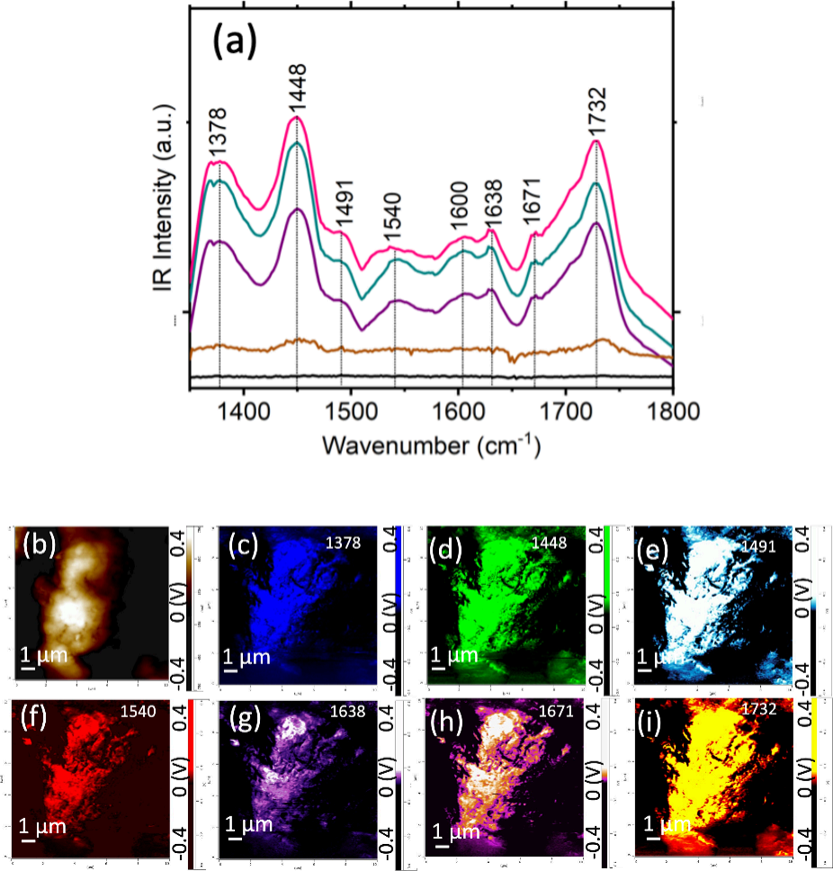
AFM-IR spectra acquired from the surface of WS_2_@PdNPs decorated with 4-NTP (a), AFM image of WS_2_@PdNPs (b) and chemical images (c-i) of WS_2_@PdNPs revealing localization of 4-NTP and DMAB.

To further investigate photocatalytic reactivity of WS_2_ and WS_2_@PdNPs, we quantified the yield of DMAB at different power of λ = 633 nm laser light (2.9 μW-140 μW), Figure S3 and Figure S4. We determined the number of TERS spectra that exhibit the vibrational signatures of 4-NTP and DMAB under each of these experimental conditions. Our results show that with an increase in the laser power, the yield of DMAB increased with a gradual decrease in the concentration of 4-NTP on the nanoplate surfaces, Figure 5, a-b. It should be noted that a nearly linear relationship between the yield of DMAB and the laser power was observed for the WS_2_ nanoplates. However, a rapid decline in the concentration of 4-NTP on WS_2_@PdNPs was observed with an increase in the lase power. We also observed no changes in the concentration of both 4-NTP and DMAB on WS_2_@Pd above 60 μW of λ = 633 nm light. Comparison of the yield of DMAB on WS_2_@PdNPs and on the WS_2_ nanoplates indicates that at low laser powers (2.9–20 μW), comparable yield of DMAB was observed on both nanostructures. However, above 20 μW, WS_2_@PdNPs demonstrated far more superior yield od DMAB compared to WS_2_, Figure 5, c-d.

**Figure 5:**
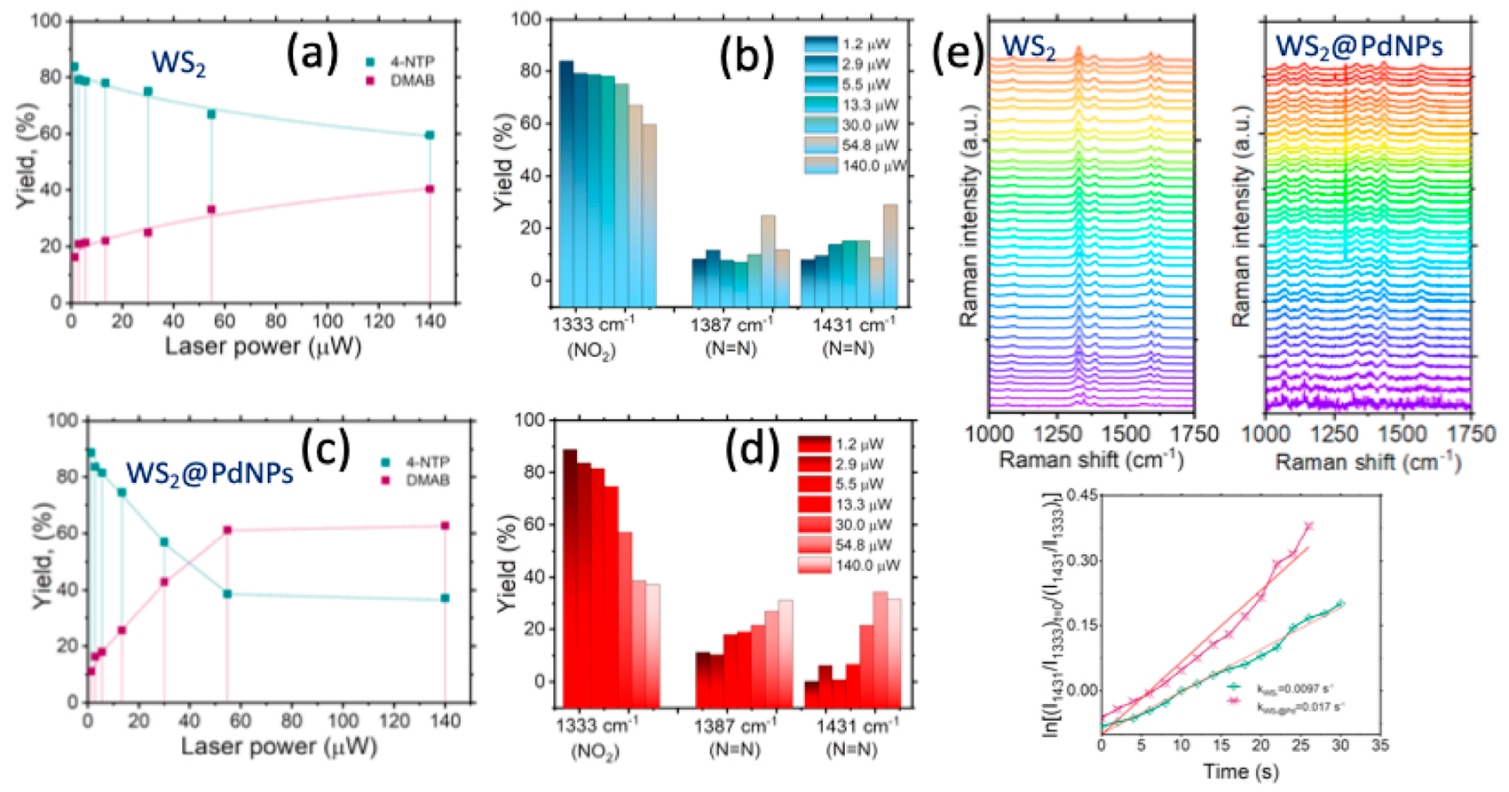
Graphs (a and c) and histograms (b and d) of changes in the concentration of 4-NTP and DMAB on the surface of WS_2_ (a and b) and WS_2_@Pd (c and d) at different powers of λ = 633 nm laser light. (e) Waterfall maps of 4-NTP to DMAB reduction on the surface of WS_2_ and WS_2_@Pd nanoplates. Rate constants of 4-NTP to DMAB reduction are based on the intensity ratio of the bands of 1431 cm^–1^ (DMAB) and 1330 cm^–1^ (NO_2_) in TERS spectra obtained from WS_2_ and WS_2_@Pd with 4-NTP on their surfaces. Each trace was fitted with a linear model according to eq 1.

Finally, we performed kinetic measurements of 4-NTP to DMAB conversion to determine the rate of photocatalytic reactions on WS_2_ and WS_2_@PdNPs. For this, we determined a change in intensities of 1431 cm^–1^ (DMAB) vs 1330 cm^–1^ (4-NTP) and plotted a natural logarithm of their ratio at different points, eq 1, Figure 5, e.^35^ We found that an increase in the light intensity causes the increase in the reaciton rate of 4-NTP to DMAB conversion on both WS_2_ and WS_2_@Pd nanoplates. Our results show that *k*_WS2_ = 0.0097 s^–1^, whereas *k*_WS2@Pd_ = 0.017 s^–1^. Thus, although rates of photoconversion of 4-NTP to DMAB were very similar on WS_2_ nanoplates, WS_2_@Pd hybrids demonstrated higher rates of photocatalysis compared to unmodified 2D dichalcogenides. This first-order kinetic relationship observed for DMAB formation on WS_2_ nanoplates and WS_2_@PdNPs hybrids suggests that the their surfaces could possess both covalently (Au–S) anchored 4-NBT and an excess of unbound 4-NBT.^27^ In this case, the rate of the reaction is determined by covalently bound 4-NBT. Thus, the dimerization of 4-NBT is a pseudo first-order reaction. These results also indicate that molecular orientation on the surface of both WS_2_ and WS_2_@PdNPs could play an important role in the catalytic reactivity of these nanostructures.
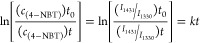
1where *c*(4-NTP)_t0_ and *c*(4-NTP)_t_ represent the concentration of 4-NTP at different reaction time. *I*_1431_ and *I*_1330_ are the intensities of the bands at 1431 cm^–1^(DMAB) and 1330 cm^–1^(NO_2_), respectively. *k* is the rate constant, and *t* is the reaction time.

Both the Brus and Jain laboratories demonstrated that illumination of noble metal nanostructures by light results in the appearance of a steady-state charge of their surfaces.^28−31^ The charge originates from unequal rates of dissipation of hot electrons and holes from the metal surface. This asymmetry results in an accumulation of hot carriers with lower transfer rates between the nanostructures and the surrounding medium. Using benzonitrile as a molecular reporter, our group quantified the magnitude of the steady-state charge on the surface of mono- and bimetallic nanostructures.^32^ Using TERS, we also demonstrated that the magnitude of the charge, and consequently reactivity of these nanostructures, could be altered by the intensity of the laser light.^32^ These results are in a good agreement with experimental and theoretical evidence reported by Jain’s group according to which a steady-state potential can be viewed as the driving force of chemical reactions.^29−31^

Illumination of 2D dichalcogenides results in the formation of excitons and trions,^33−35^ which, in turn can dissipate forming a steady-state charge on the surface of WS_2_ and WS_2_@Pd nanoplates. We hypothesize that this steady-state charge, also described as photopotential by the Lagugné-Labarthet group,^19^ can drive the dimerization of 4-NTP into DMAB. Our results also suggest that PdNPs confine the charge which results in the superior reactivity of WS_2_@PdNPs compared to WS_2_. Alternatively, excitons formed on WS_2_ and WS_2_@Pd nanoplates can directly trigger the observed chemical transformation in 4-NTP. Additional studies are required to disentangle these two possible catalytic mechanisms. This work is currently in progress in our group.

## Conclusion

Our results indicate that light-induced excitons on WS_2_ and WS_2_@PdNPs coupled were capable of reducing 4-NTP into DMAB. We also found that central parts of both WS_2_ and WS_2_@Pd nanoplates exhibit much stronger photocatalytic properties compared to their edges. Using TERS, we were able to determine the relationship between light power, rate, and yields of photocatalytic reactions on the surface of WS_2_ and WS_2_@PdNPs. Our results indicate that WS_2_@PdNPs demonstrate a very similar yield of DMAB at low laser powers (2.9–20 μM). However, a superior yield of DMAB was observed on WS_2_@Pd compared to WS_2_ nanoplates at the laser powers above 20 μM. Thus, our results indicate that catalytic performance of WS_2_ nanoplates could be enhanced by Pd nanoparticles.

## Supporting Information Available

Additional experimental details, materials, and methods are summarized in Supporting Information. Figure S1 shows TER spectra of WS_2_@PdNPs; Figure S2 summarizes results of AFM-IR imaging of the surface of WS_2_nanoplates coated by 4-NTP. Figure S3 and S4 show TERS spectra acquired from WS2 and WS2@PdNPs modified by 4-NTP at different laser light intensities (PDF)
