# Supplementary material for: Nanoscale Imaging of Palladium-Enhanced Photocatalytic Reduction of 4-Nitrothiophenol on Tungsten Disulfide Nanoplates
Source: Nano Lett. 2024 Oct 7;24(41):13004–9. doi: 10.1021/acs.nanolett.4c03702 (PMC11487628; doi:10.1021/acs.nanolett.4c03702)
Supplement: Supplementary file 1 — nl4c03702_si_001.pdf [file nl4c03702_si_001.pdf]

Nanoscale Imaging of Palladium-Enhanced Photocatalytic Reduction of 4- Nitrothiophenol on Tungsten Disulfide Nanoplates

Swati Patil[1], and Dmitry Kurouski*[1,2]

[1]Department of Biochemistry and Biophysics, Texas A&M University, College Station, Texas 77843, United States
[2]Department of Biomedical Engineering, Texas A&M University, College Station, Texas, 77843, United States

Supporting Information

Experimental details

Chemicals
All chemical regents were purchased from Sigma-Aldrich (St. Lois, MO) and used without further any purification.

Modification of WS_2@PdNPs
Si wafers were first cut into small pieces around 5×5 mm each and then cleaned using acetone and ethanol. Next, pieces of Si wafers were dried under nitrogen gas. Freshly synthesized WS_2 and WS_2@PdNPs were drop-casted on the Si surface and kept at room temperature for 30 min, allowing for the adsorption of nanoparticles onto the surface. Next, 2 mM ethanolic solution of 4-NTP was applied on WS_2 and WS_2@PdNPs. After 1h of exposition, WS_2 and WS_2@PdNPs were rinsed with excessive copies of ethanol to remove unbound 4-NTP.

AFM Probe-preparation
240AC AFM tips were purchased from Opus, Watsonville, CA. The scanning probes had a force constant of 2 N/m, resonance frequency of 70 kHz, and an amplitude of 20 nm. Next, AFM tips were placed in a

thermal evaporator (MBrown, Stratham, NH) and coated with 70 nm of gold (Kurt J. Lesker, Efferson Hills, PA) at 0.1 A/s rate. Temperature at the tip surface was ~54 °C upon the metal deposition.

TERS Imaging

Nanoscale imaging was performed using AIST-NT AFM-TERS system equipped with 633 nm continuous wavelength (CW) laser. Laser power was adjusted using neutral density filter wheel. The laser was focused on the sample at 45 ° angle using 100× Mitutoyo microscope objective. The same objective was used to collect scattered light that was directed to HORIBA iHR550 spectrometer equipped with a Synapse EM-CCD camera (HORIBA, Edison, NJ). Prior to entering the spectrograph, dichroic mirror was used to remove elastically scattered photons.

Nano-Infrared spectroscopy

Imaging and spectral analysis were performed on nanoIR3 system (Bruker, Santa Barbara, CA, USA), equipped with a QCL laser. ContGB-G AFM contact mode scanning probes were utilized (resonance frequency of 13 kHZ, spring constant of 0.2 N/m, and a length of 450 µm). The tip was first optimized using a polymethyl acrylate standard for the wavenumbers: 1400-1800 cm^{-1}. Laser parameters include a power of 25.49%, polarization at 90 degrees, IR focus of 70456, and a pulse rate around 828 kHz. Images were acquired at a scan rate of 0.5-0.8 Hz, an I and P gain ranging from 1/2 to 5/10, and a resolution of 256 for both the X and Y. A total of 30 spectra per sample were obtained with a co-average of 3 for each spectrum at a spectral resolution of 2 cm^{-1}/pt.

The spectra were zapped at the 1648-1652 points to remove an artifact caused by the chip-to-chip transition of the laser at this region. Spectral processing was conducted using MATLAB, equipped with a PLS Toolbox version 9.0 (Eigenvector Research, Inc., Manson, WA). Spectra are first applied a smoothing processing of Savitzky-Golay at a polynomial order of 0, area normalized and baselined with automated weighted least squares.

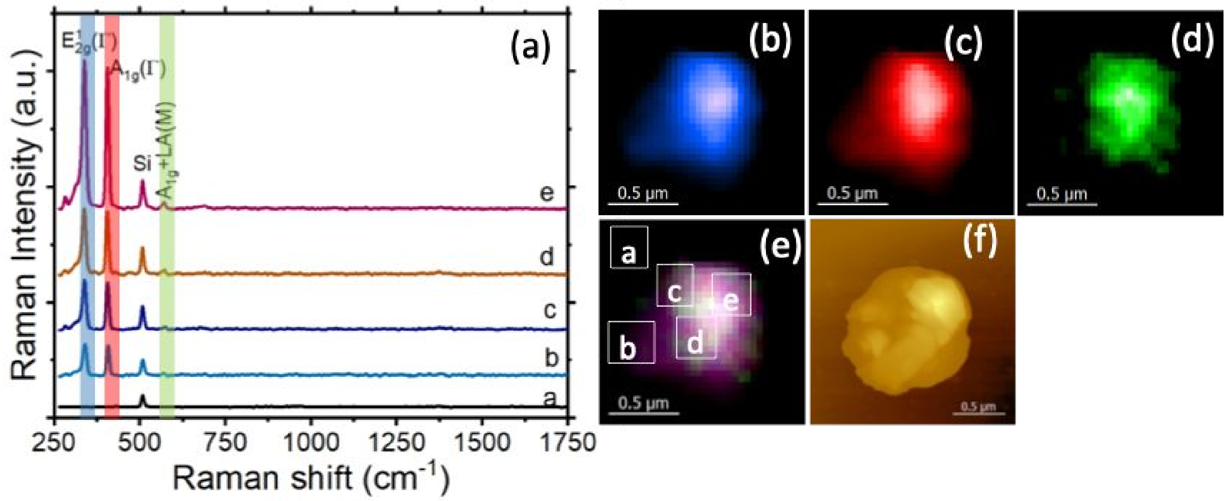

Figure S1. (a) TER spectra of WS_2@PdNPs. (b-e) TERS image of WS_2@PdNPs with E_{2g} (blue), A_{1g}(green) and A_{1g} +LA(M)(red) bands. (e) TERS image of WS_2@PdNPs from overlapping E_{2g}, A_{1g} and A_{1g}+LA(M)vibrations. (f) corresponding AFM image of WS_2@PdNPs.

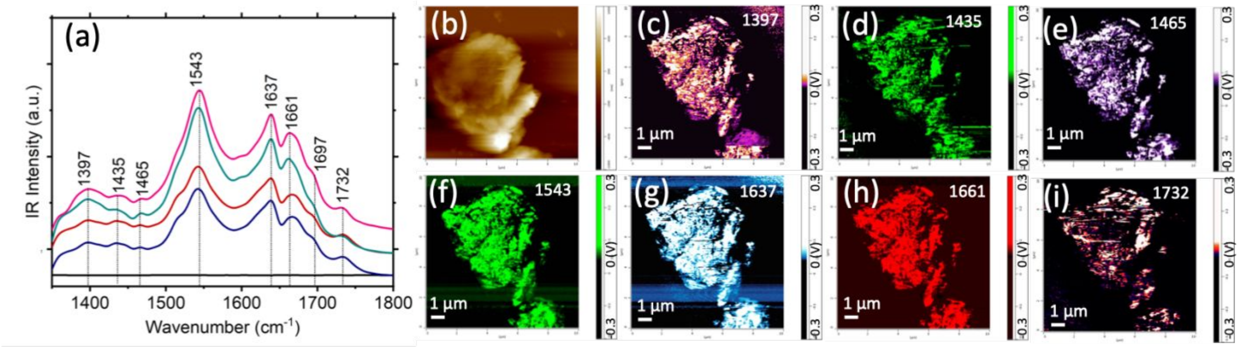

Figure S2. AFM-IR spectra acquired from the surface of WS_2 nanoplates coated by 4-NTP (a), AFM image of WS_2 nanoplates (b) and chemical images (c-i) of WS_2 nanoplates revealing localization of 4-NTP and DMAB.

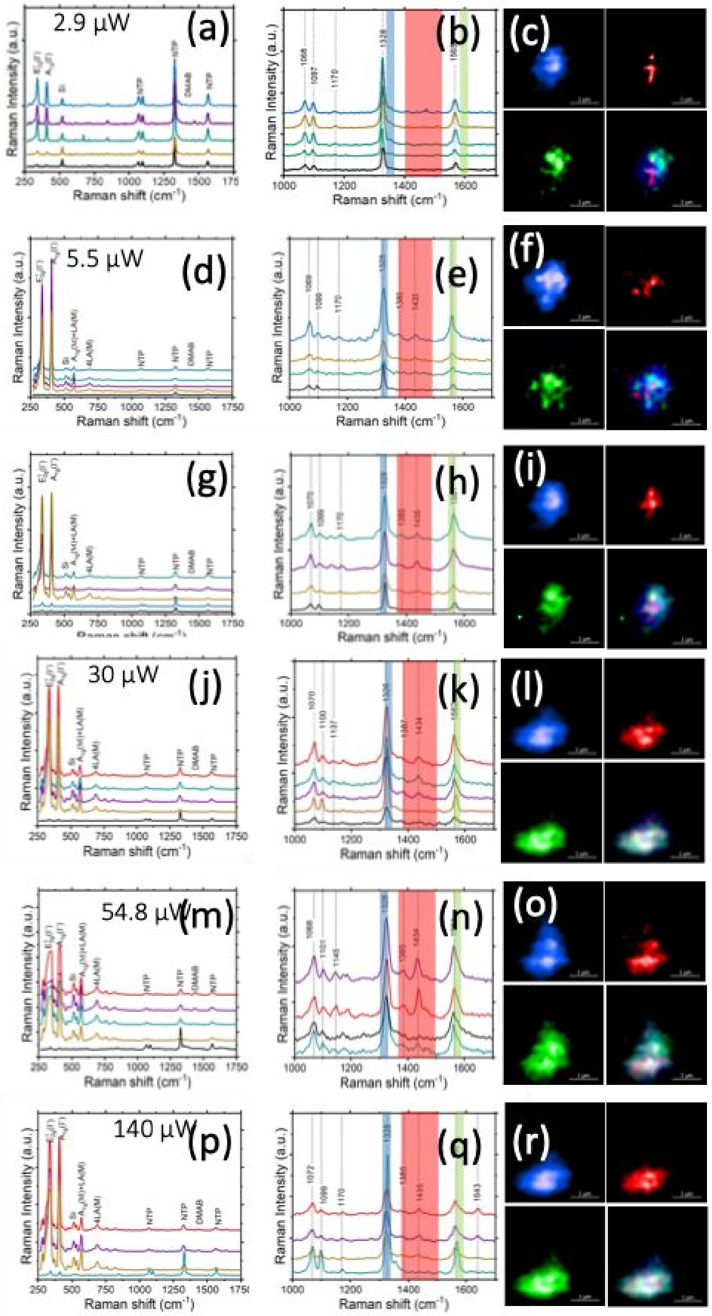

Figure S3. (a, d, g, j, m, p) TER spectra extracted from chemical maps of WS_2 nanoplate modified by 4-NTP. (b, e, h, k, n, q) The catalytic reaction is clearly showing product and reactants with observed highly intense peaks of 4-NTP and DMAB on WS_2 nanoplate. (c, f, i, l, o, r) TERS image of WS_2 with (c) and (e) 4-NTP and (d) DMAB. (f) TERS image of WS_2 from overlapping 4-NTP and DMAB. The scanning step size was 10 nm per pixel, spectral acquisition time was 0.5 s.

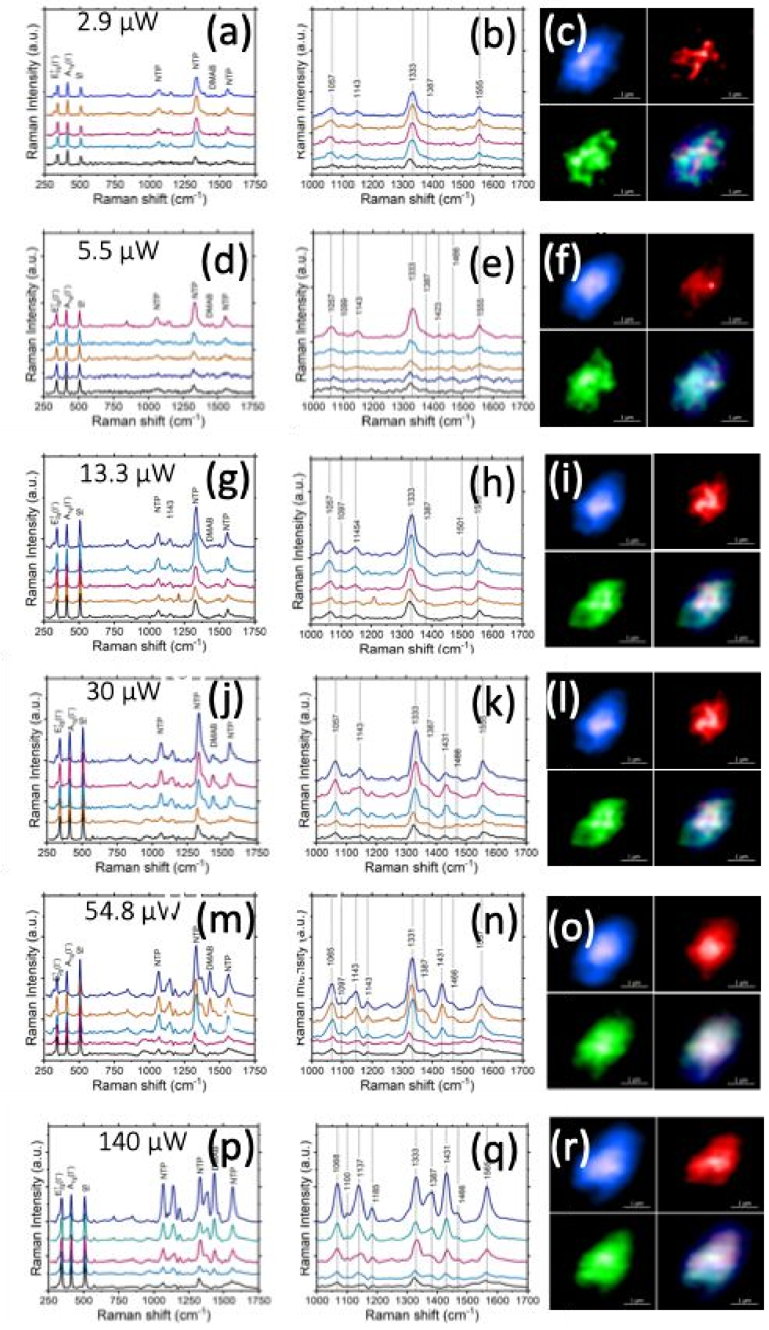

Figure S4. (a, d, g, j, m, p) TER spectra extracted from chemical maps of WS_2@PdNPs modified by 4-NTP. (b, e, h, k, n, q) The catalytic reaction is clearly showing product and reactants with observed highly intense peaks of 4-NTP and DMAB on WS_2 nanoplate. (c, f, i, l, o, r) TERS image of WS_2 with (c) and (e) 4-NTP and (d) DMAB. (f) TERS image of WS_2 from overlapping 4-NTP and DMAB. The scanning step size was 10 nm per pixel, spectral acquisition time was 0.5 s.
